# Supplementary material for: Long non-coding RNA CCRR controls cardiac conduction via regulating intercellular coupling
Source: Nat Commun. 2018 Oct 9;9:4176. doi: 10.1038/s41467-018-06637-9 (PMC6177441; doi:10.1038/s41467-018-06637-9)
Supplement: Supplementary file 1 — Supplementary Information [file 41467_2018_6637_MOESM1_ESM.pdf]

# Long non-coding RNA CCRR controls cardiac conduction via regulating intercellular coupling

**Zhang *et.al.***

## Supplementary Figures

## Supplementary Figures

### Supplementary Figure 1

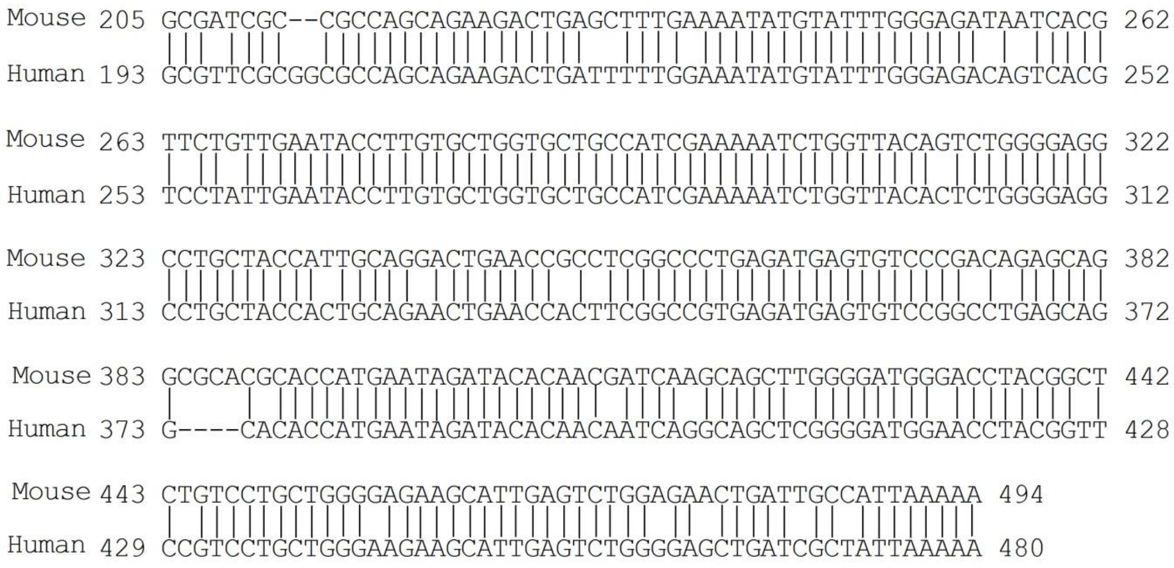


**Supplementary Figure 1. Alignment of the CCRR sequences between mouse and human showing the species conservation.** The conserved regions are indicated by "|".

**Supplementary Figure 2**

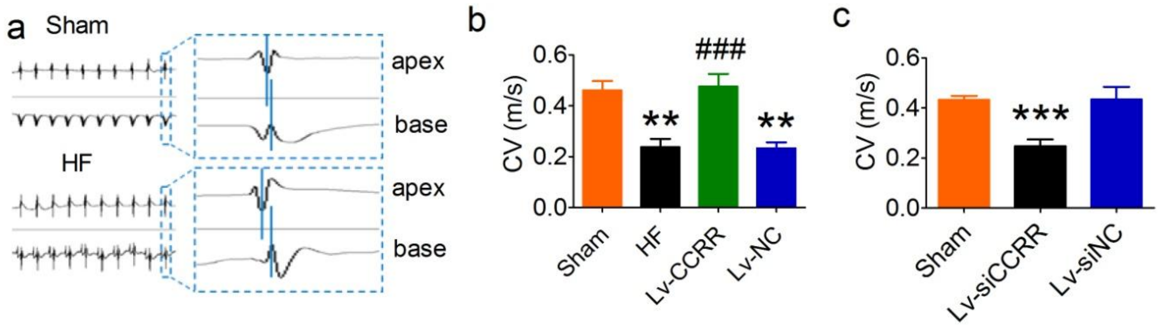


**Supplementary Figure 2. Regulation of cardiac conduction by lncRNA-CCRR in a mouse of model of HF.** (**a**) Cardiac conduction velocity (CV) was measured by cardiac activation captured by surface electrocardiogram (ECG) simultaneously recorded at apex and base of Langendorff-perfused mouse hearts. Shown are representative traces of ECG. The distance (D) between the two recording electrodes was fixed to 1 cm. The vertical lines indicate the second detectable notch of the recorded QRS waves and the time-lag (ΔT) of the vertical lines between the two locations indicate the difference of the activation time between apex and base or the time required for the excitation wave to conduct/propagate from apex to base. (**b**) Slowing of CV and restoration by CCRR overexpression in HF mice. Shown are the averaged data of CV calculated by dividing the distance by the time-lag ($CV = D/\Delta T$). $^{**}P<0.01$ HF *vs*. Sham Control, $^{###}P<0.001$ Lv-CCRR *vs*. HF, $^{**}P<0.01$ Lv-NC *vs*. Sham Control; n=6. Note the increased lag time in HF (lower left) compared with that in the Sham control (upper left), indicating a slowing of CV in HF, and this conduction slowing was restored in the hearts pretreated with the lentivirus carrying the CCRR gene for overexpression (Lv-CCRR), but not with the negative control construct (Lv-NC). Viral vectors were administered by intra-cavity injection (directly injected into the left ventricular chamber). (**c**) Slowing of CV induced by CCRR knockdown in healthy mice. The lentivirus vector engineered to contain a CCRR siRNA (Lv-siCCRR) was injected into the left

ventricular chamber to silence myocardial CCRR. Lv-siCCRR caused a remarkable decrease in cardiac CV, whereas the negative control (Lv-siNC) failed to elicit any significant changes. ***$P$<0.001 Lv-siCCRR *vs*. Sham Control; n=7. (Mean ±SEM; analysis of variance-ANOVA followed by Dunnett's test for comparisons among multiple groups, and Student *t*-test for comparisons between two groups).

**Supplementary Figure 3**

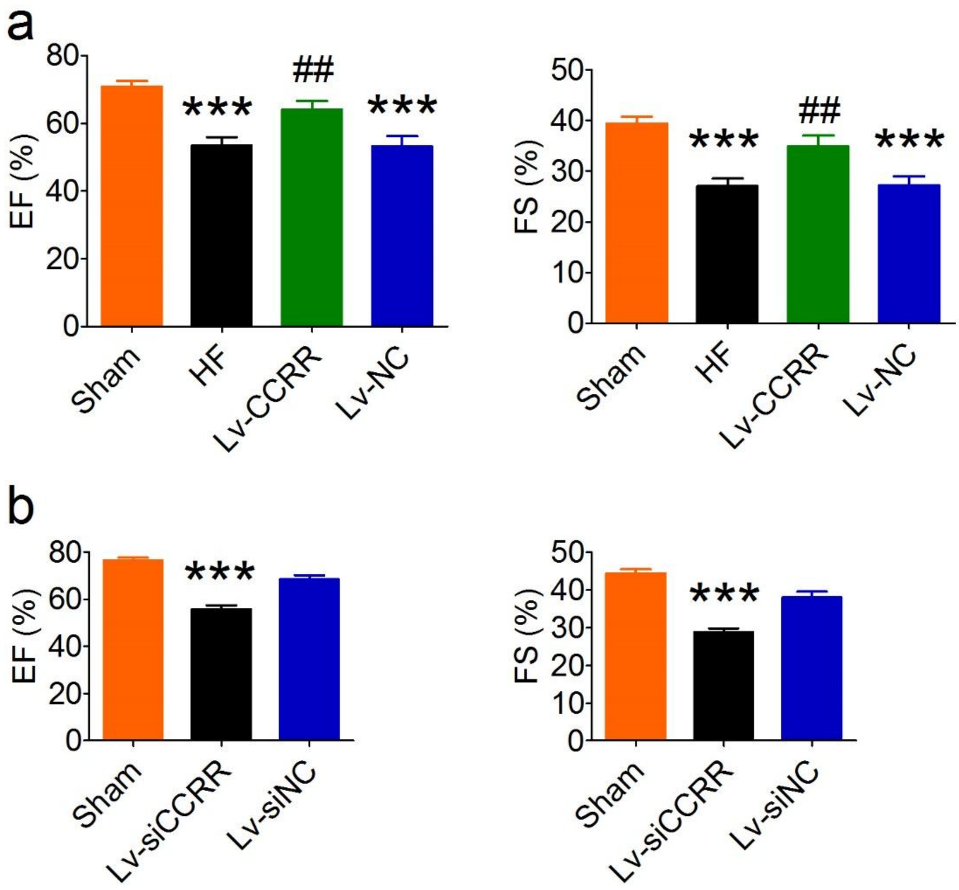


**Supplementary Figure 3. Effects of CCRR on cardiac contractile function.** (**a**) Impaired cardiac function in HF and restoration by CCRR overexpression. Cardiac function, including ejection fraction (EF) and fractional shortening and other parameters (see **Supplementary Tables 2 & 3**), was measured by echocardiography. HF rendered considerable reduction of both EF and FS, and these deleterious alterations were tremendously abrogated by Lv-CCRR. ****P*<0.001 HF (n=10) or Lv-NC (n=10) *vs*. Sham control (n=10); ##*P*<0.01 Lv-CCRR (n=17) *vs*. HF. (**b**) Depression of cardiac function induced by CCRR knockdown in healthy mice (also see **Supplementary Table 4**). ****P*<0.001 Lv-siCCRR *vs*. Sham Control; n=26. (Mean ±SEM; ANOVA followed by Dunnett's test for multiple group comparisons, and Student *t*-test for two group comparisons).

**Supplementary Figure 4**

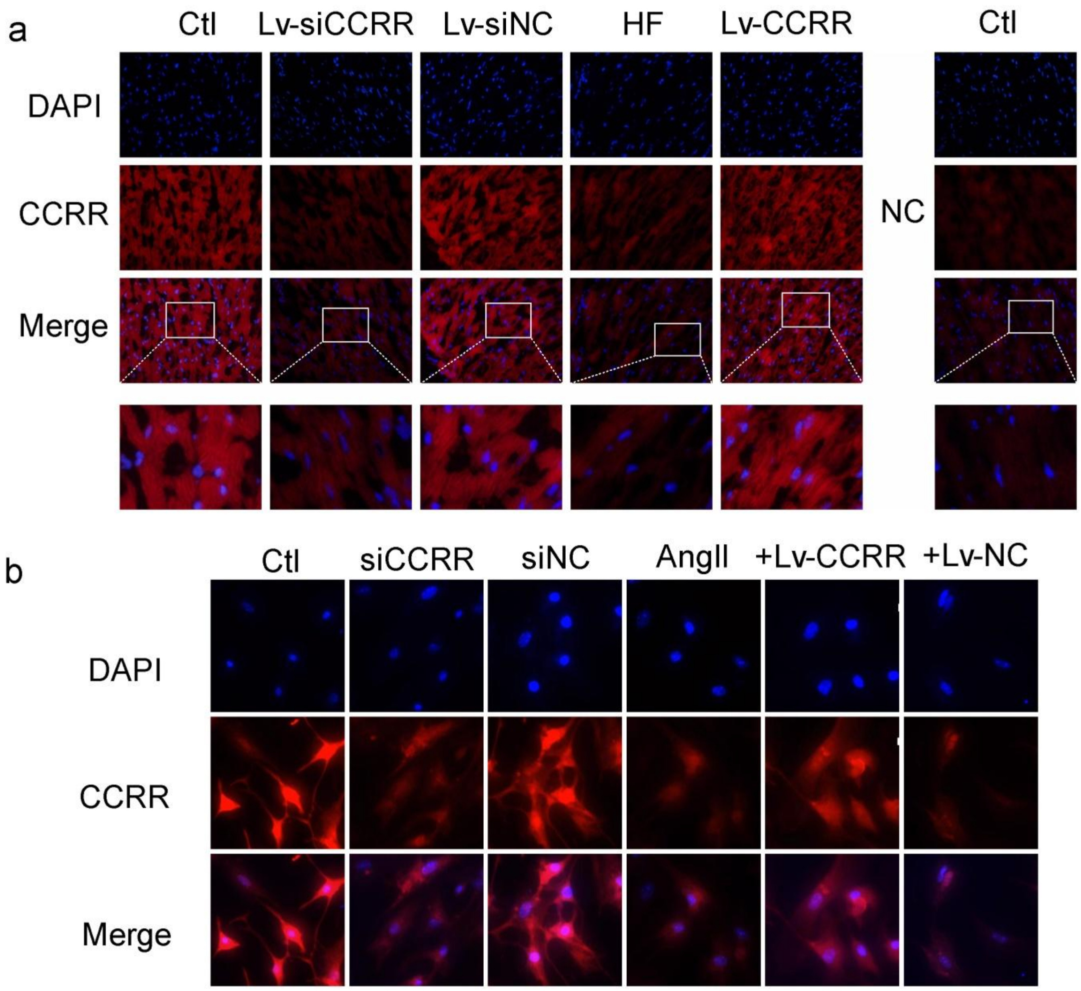


**Supplementary Figure 4. Comparisons of CCRR levels detected by fluorescence in situ hybridization (FISH) under varying conditions.** (**a**) FISH images showing the changes of CCRR expression and subcellular distribution in the cardiac sections of HF mice pretreated with varying constructs. Similar results were observed in another 2 experiments. (**b**) FISH images showing the changes of CCRR expression and subcellular distribution in vitro in cultured neonatal mouse ventricular myocytes (NMVMs) pretreated with varying constructs, +Lv-CCRR: AngⅡ+Lv-CCRR; +Lv-NC: AngⅡ+Lv-CCRR. Similar results were observed in another 5 experiments.

## Supplementary Figure 5

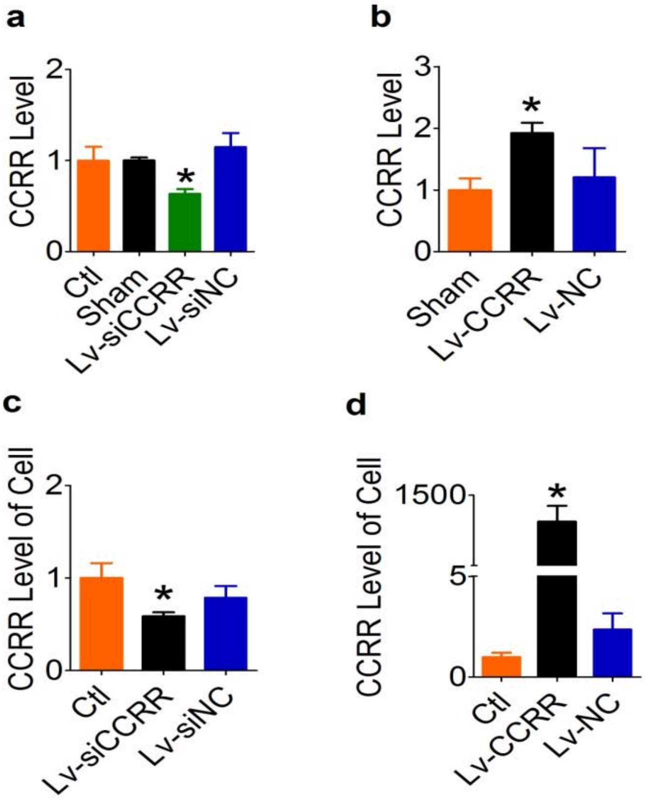


**Supplementary Figure 5. Verification of efficiency of lentivirus-mediated CCRR overexpression and lentivirus-mediated silencing of CCRR in myocardium or in cultured cardiomyocytes.** (**a**) Knockdown of endogenous CCRR by the lentivirus vector carrying a CCRR siRNA (Lv-siCCRR) in mouse myocardium. *$P$<0.05 *vs.* Sham; n=10 mice. (**b**) Overexpression of CCRR by the lentivirus vector carrying the CCRR gene (Lv-CCRR) in mouse myocardium. *$P$<0.05 *vs.* Sham; n=3 mice. (**c**) Knockdown of CCRR by Lv-siCCRR treatment in cultured neonatal mouse ventricular myocytes (NMVMs). *$P$<0.05 *vs.* control (Ctl); n=5 batches of NMVMs. (**d**) Overexpression of CCRR by Lv-CCRR treatment in NMVMs. *$P$<0.05 *vs.* control (Ctl); n=3 batches of NMVMs. (Mean ±SEM; analysis of variance—ANOVA followed by Dunnett's test for comparisons among multiple groups, and Student *t*-test for comparisons between two groups).

**Supplementary Figure 6**

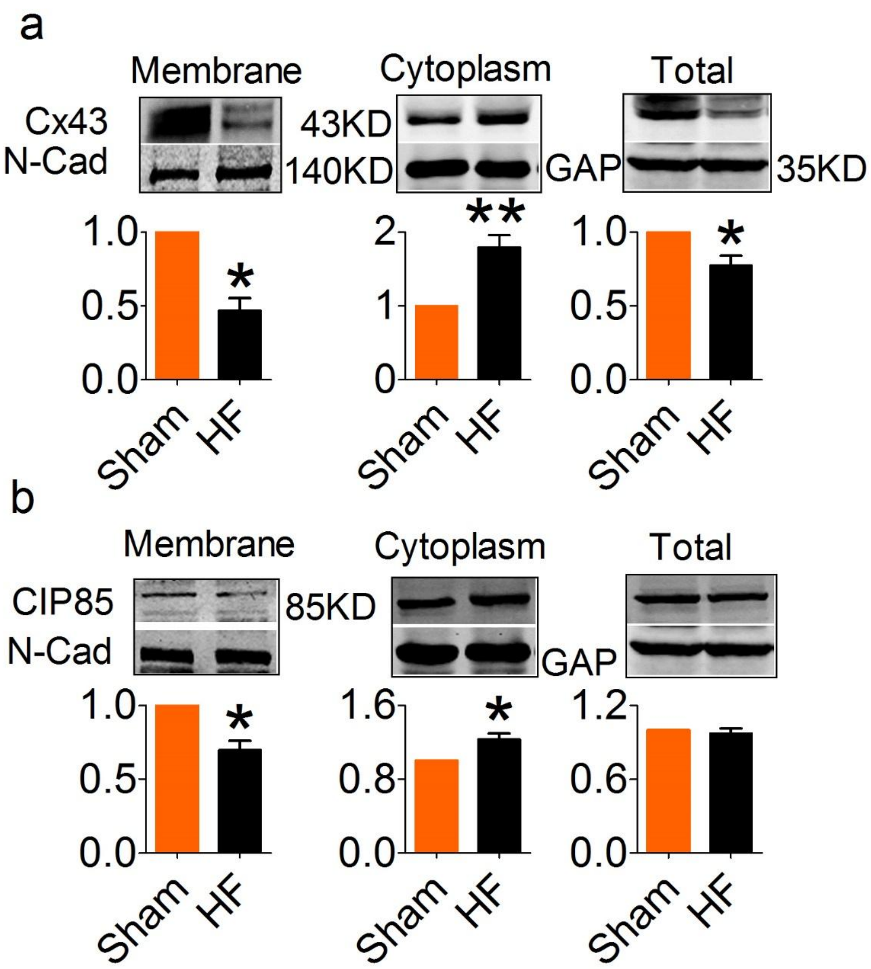


**Supplementary Figure 6. Expression alterations of connexin43 (Cx43) and CIP85 (the Cx43-interacting protein that regulates the endocytic trafficking of Cx43) in a mouse model of heart failure (HF) induced by pressure-overload.** Comparisons of Cx43 and CIP85 at the protein levels between the membrane and cytoplasm fractions. Note that Cx43 levels were prominently decreased in the membrane (**P*<0.05 HF *vs*. Sham; n=3 mouse hearts), but increased in the cytoplasm (***P*<0.01 HF *vs*. Sham; n=5), which resulted in a net downregulation of the total protein level (**P*<0.05 HF *vs*. Sham; n=7), indicating the enhanced endocytic trafficking of Cx43 in HF mice compared to the sham-operated control animals. Similar changes were observed with CIP85. CIP85 levels were prominently

decreased in the membrane (*$P$<0.05 HF *vs*. Sham; n=3), but increased in the cytoplasm (*$P$<0.05 HF *vs*. Sham; n=4), which resulted in a net unaffected of the total protein level (HF *vs*. Sham; n=7). The membrane bands were normalized to N-cadherin (N-Cad), and the cytosolic and total protein bands were normalized to GAPDH. (Mean ±SEM; analysis of variance—ANOVA followed by Dunnett's test for comparisons among multiple groups, and Student *t*-test for comparisons between two groups).

**Supplementary Figure 7**

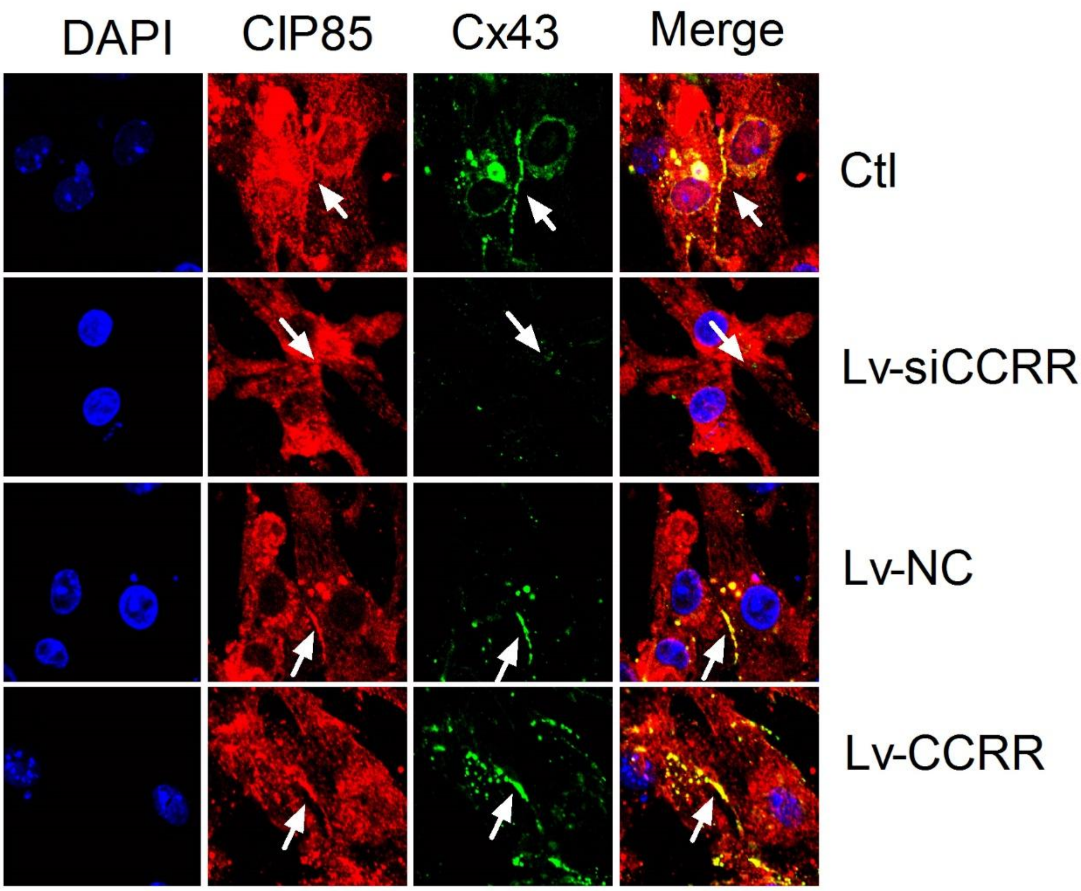


**Supplementary Figure 7. Immunocytostaining of CIP85 and Cx43 in cultured neonatal mouse ventricular myocytes (NMVMs) pretreated with varying constructs.** Similar observations were consistently obtained from another 3 batches of cells. Note that knockdown of CCRR by Lv-siCCRR reduced, whereas overexpression of CCRR by Lv-CCRR increased, the presence of Cx43 in the plasma membrane.

**Supplementary Figure 8**

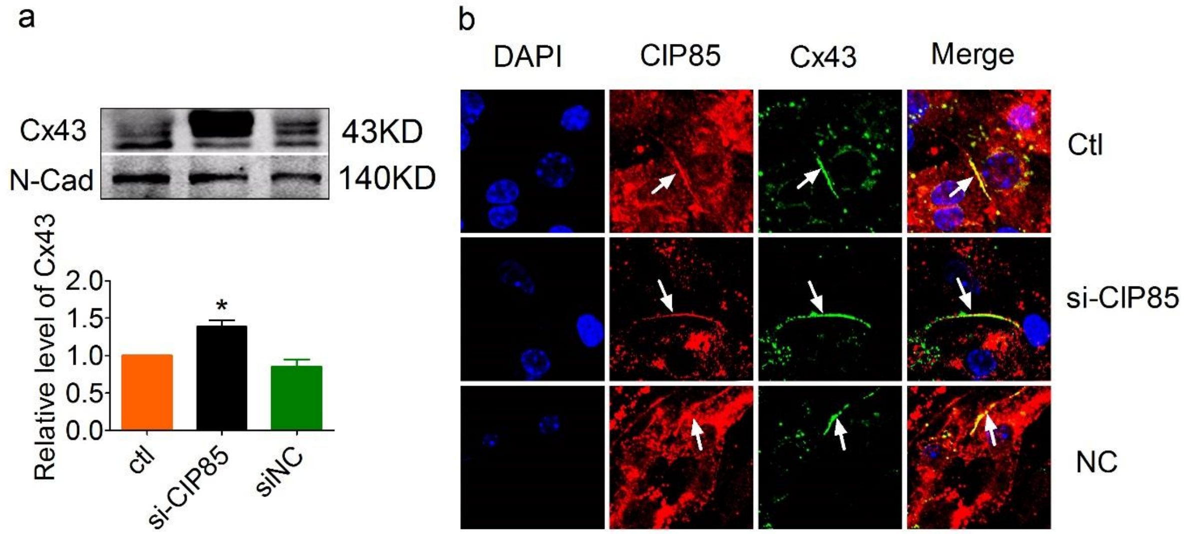


**Supplementary Figure 8. Evidence for the interaction between CIP85 and Cx43 as reported by immunoblotting and immunocytostaining analyses cultured neonatal mouse ventricular myocytes (NMVMs).** (**a**) Effect of silencing CIP85 by siRNA (si-CIP85) on the expression of Cx43 in the plasma membrane. Note that knockdown of CIP85 increased Cx43 protein level in the membrane. Upper panel: a typical example of Western blot bands. Lower panel: Averaged values of protein band densities. siNC: scrambled negative control siRNA. **P*<0.05 *vs*. Ctl and siNC; n=3. (Mean±SEM; analysis of variance—ANOVA followed by Dunnett's test for comparisons among multiple groups, and Student *t*-test for comparisons between two groups). (**b**) Representative images of immunocytostaining showing the enhancing effect of si-CIP85 on Cx43 localization on the cytoplasmic membrane. Similar results were obtained from another 3 independent experiment.

**Supplementary Figure 9**

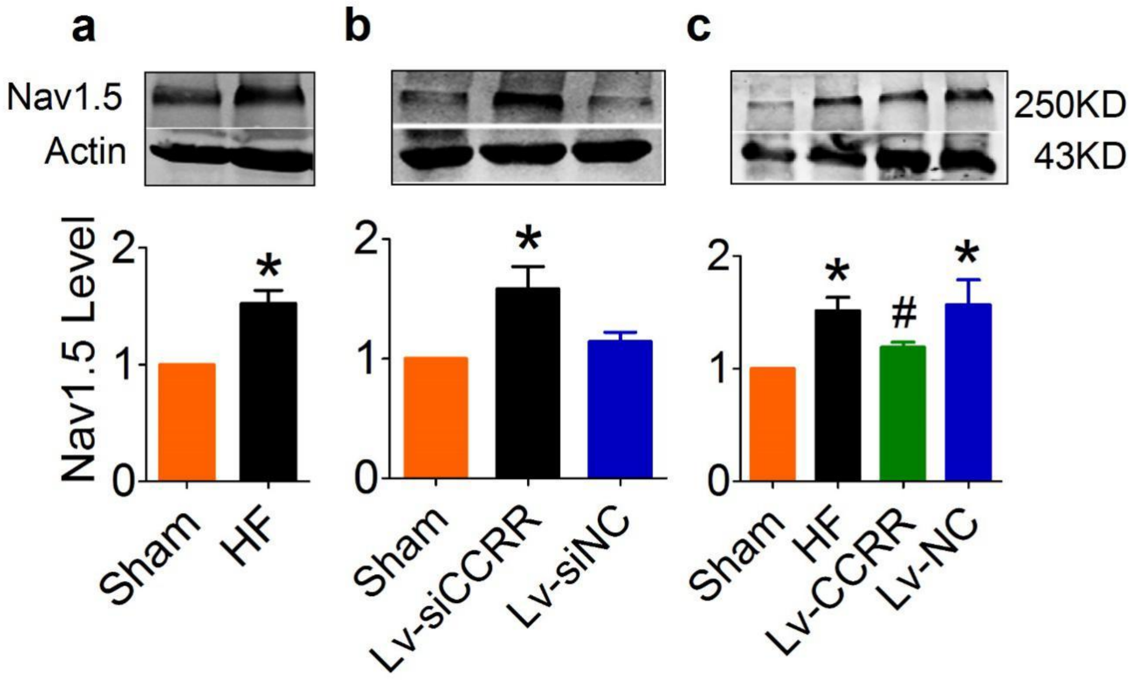


**Supplementary Figure 9. Expression alterations of Nav1.5 in healthy mice pretreated with Lv-siCCRR to knockdown endogenous CCRR and HF mice pretreated with Lv-CCRR for CCRR overexpression.** (**a**) Nav1.5 levels were prominently increased (total protein level) in the myocardium of HF mice compared to the sham-operated control animals (Sham). **P*<0.05 HF *vs*. Sham; n=5 mouse hearts. (**b**) Effects of CCRR knockdown by Lv-siCCRR on the protein levels of Nav1.5 in the myocardium of healthy mice. **P*<0.05 Lv-siCCRR *vs*. Sham control; n=9. (**c**) Effects of CCRR overexpression by Lv-CCRR on the protein levels of Nav1.5 in the myocardium of HF mice. Upper panel: examples of Western blot bands; lower panel: averaged data on Nav1.5 protein levels. **P*<0.05 HF or Lv-NC*vs*. Sham; [#]*P*<0.05 Lv-CCRR *vs*. HF; n=5.

**Supplementary Figure 10**

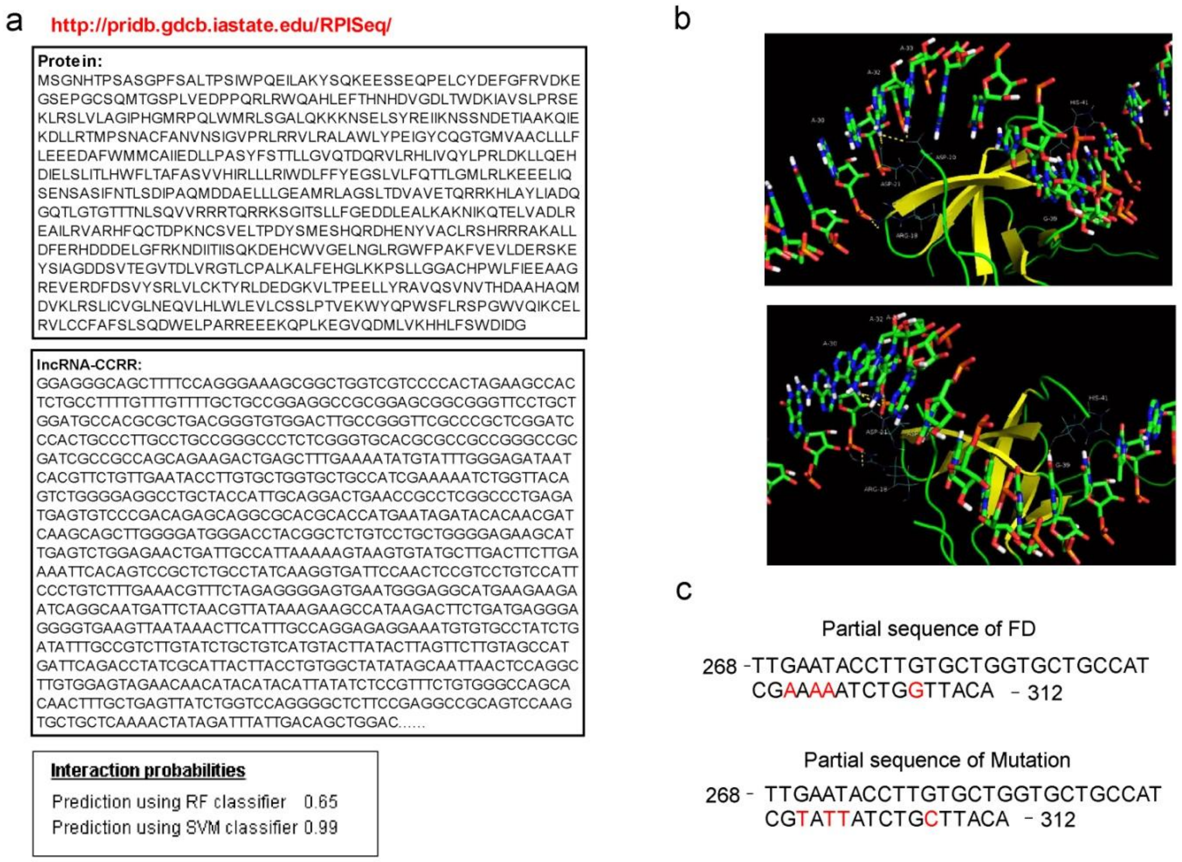


**Supplementary Figure 10.** (**a**) Theoretical analysis of RNA:protein binding using the RNA-Protein Interaction Prediction (RPISeq) database, which revealed a high probability of CCRR:CIP85 interaction. (**b**) Structural cartoons showing the computational docking between CCRR and FD (the putative functional domain of lncRNA CCRR represented by sticks) and CIP85 protein (represented by ribbons) by Hex 8.0. Hex 8.0 is an interactive protein docking and molecular superposition program for calculating and displaying feasible docking modes of pairs of protein and nucleic acid molecules (http://www.loria.fr/~ritchied/hex/)13. Upper panels give top views and lower panels show the side views. (**c**) Partial sequence of FD showing the exact sites of nucleotide replacement mutation (red) to the predicted binding site for CIP85.

**Supplementary Figure 11**

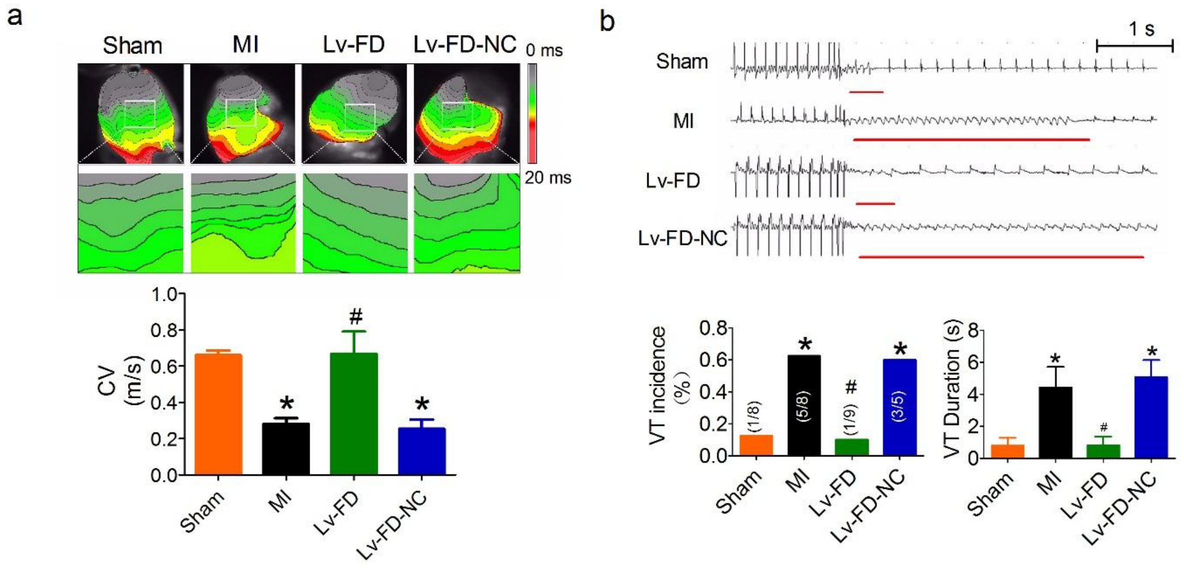


**Supplementary Figure 11. Functional role of the conserved sequence motif (FD for Functional Domain) of CCRR in a mouse model of myocardial infarction (MI).** (**a**) Slowing of conduction velocity (CV) in MI and restoration by FD overexpression. CV was determined by optical mapping techniques with a voltage-sensitive dye to define the cardiac activation. CV values were calculated from the gradient of the scalar field of 12-ms isochronal activation maps along the septal apex-base axis: CV = distance/12 ms. Note that CV was substantially decreased in MI and this conduction slowing was restored in the hearts pretreated with the lentivirus carrying FD for overexpression (Lv-FD), but not with the negative control construct (Lv-FD-NC). Viral vectors were administered by intra-cavity injection (directly injected into the left ventricular chamber). The Sham group underwent the same surgical procedures with LAD occlusion. *$P$<0.05 MI or Lv-NC *vs*. Sham control; $^{\#}P$<0.05 Lv-FD *vs*. MI; n=3. (**b**) Anti-arrhythmic effects of FD overexpression in a mouse model of MI. The incidence and duration of ventricular tachycardia (VT) induced by programmed stimuli were determined from ECG recordings. Note that the ischemic arrhythmogenicity was significantly enhanced in MI hearts, which was considerably

suppressed by Lv-FD but not by Lv-FD-NC. The red lines in the ECG traces indicate VT duration, and the values within the parentheses in the bar charts indicate VT incidence. *$P$<0.05 MI or Lv-FD-NC *vs*. Sham control; $^{\#}P$<0.05 Lv-FD *vs*. MI. (Mean ±SEM; ANOVA followed by Dunnett's test for multiple group comparisons, Student *t*-test for two group comparisons, and $\chi^2$-test for non-parametric data set comparisons).

**Supplementary Figure 12**

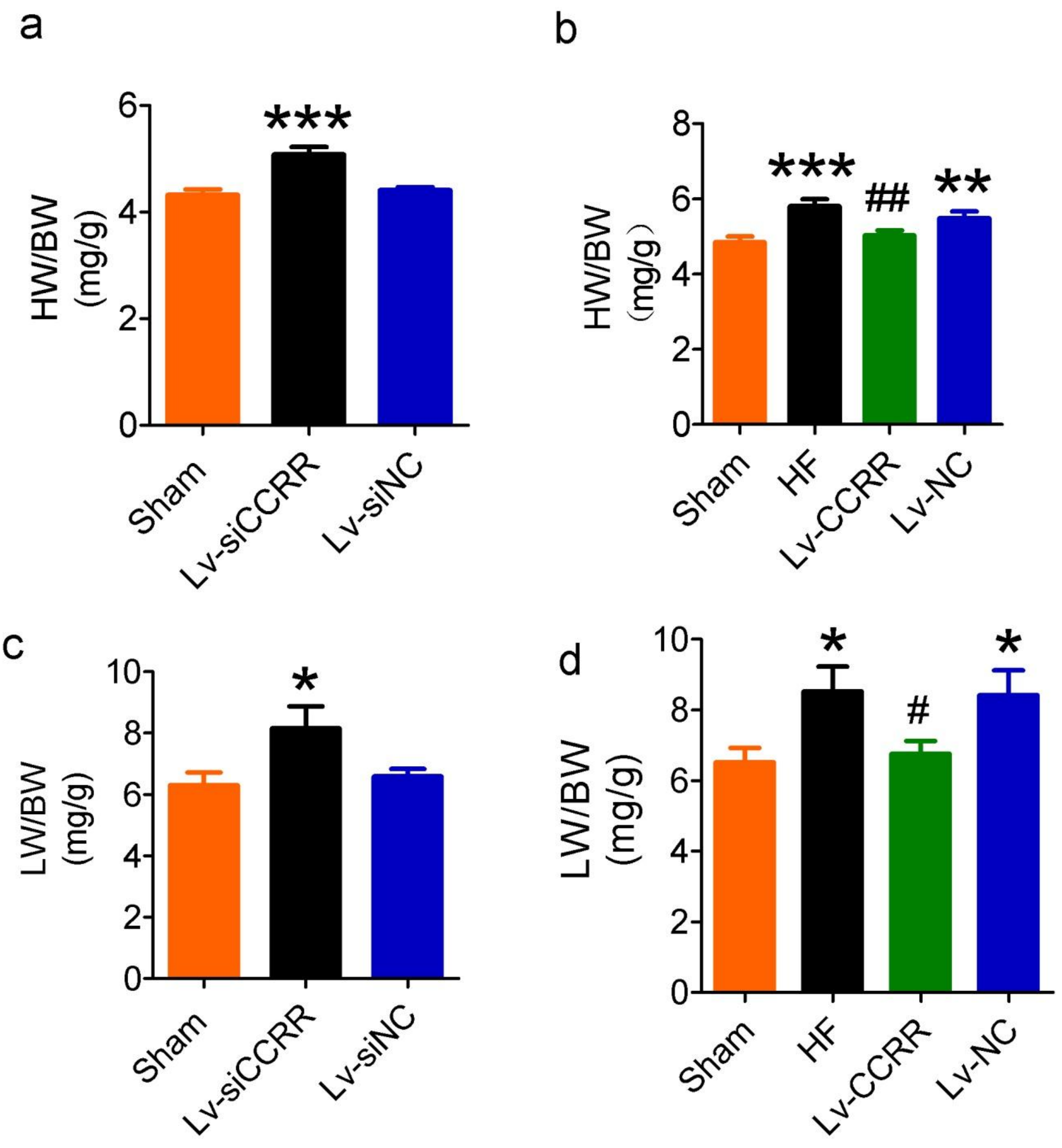


**Supplementary Figure 12. Alterations of heart weight/body weight ratio (HW/BW) and lung weight/body weight ratio (LW/BW) as evidence for the development of heart failure.** (**a**) Effect of CCRR knockdown by Lv-siCCRR on HW/BW ratio in healthy mice. Lv-siCCRR: the lentivirus vector engineered to contain a CCRR siRNA fragment; Lv-siNC: the lentivirus vector engineered to contain a negative control siRNA fragment. The constructs were injected into the left ventricular chamber. ****P*<0.001 Lv-siCCRR *vs*. Sham and

Lv-siNC; n=10 mouse hearts. (**b**) Effect of CCRR overexpression by Lv-CCRR on HW/BW ratio in a mouse model of heart failure (HF) induced by transverse aorta constriction (TAC). Lv-CCRR: the lentivirus carrying the CCRR gene for overexpression; Lv-NC: the negative control viral construct. $^{**}P<0.01$ & $^{***}P<0.001$ HF *vs*. Sham; $^{##}P<0.01$ Lv-CCRR *vs*. HF and Lv-NC; n=8. (**c**) Effect of CCRR knockdown by Lv-siCCRR on LW/BW ratio in healthy mice. $^{*}P<0.051$ Lv-siCCRR *vs*. Sham and Lv-siNC; n=6 mouse hearts. (**d**) Effect of CCRR overexpression by Lv-CCRR on LW/BW ratio in HF mice. $^{*}P<0.05$ HF *vs*. Sham; $^{#}P<0.05$ Lv-CCRR *vs*. HF and Lv-NC; n=6. (Mean±SEM; analysis of variance—ANOVA followed by Dunnett's test for comparisons among multiple groups, and Student *t*-test for comparisons between two groups).

**Supplementary Figure 13**

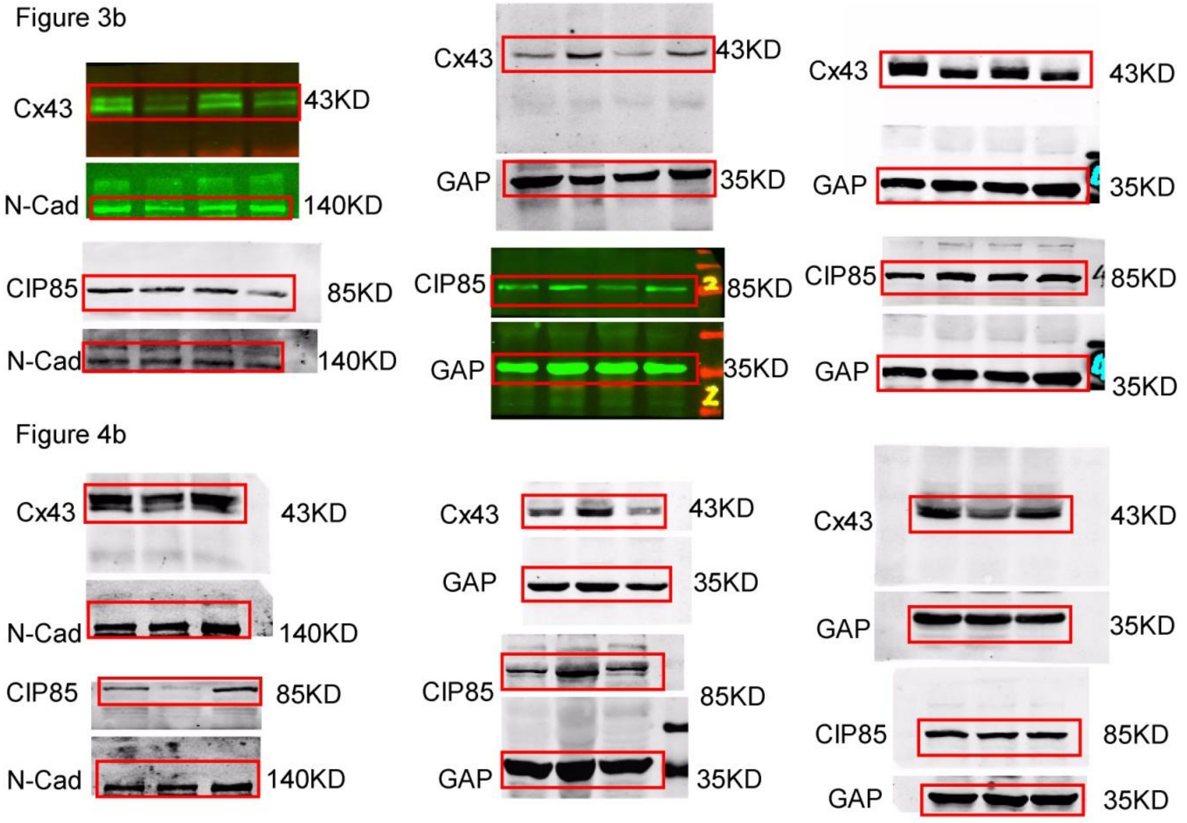


**Supplementary Figure 13. The full scans of all Western blots and gels presented in the manuscript. Figure 3b,** Effects of CCRR overexpression by Lv-CCRR on the protein levels of connexin43 (Cx43) and CIP85 (the Cx43-interacting protein that regulates the endocytic trafficking of Cx43 for degradation) in HF mice. **Figure 4b**, Effects of CCRR knockdown by Lv-siCCRR on the protein levels of Cx43 (up panel) and CIP85 (lower panel) in healthy mice, with comparisons between the membrane and cytoplasm fractions.

**Supplementary Tables**

**Supplementary Table 1. The demographic characteristics in heart failure (HF) patients and non-HF control subjects**

| | Non-HF | | | HF | | | | |
|---|---|---|---|---|---|---|---|---|
| | 1 | 2 | 3 | 1 | 2 | 3 | 4 | 5 |
| Gender | male | male | Male | Male | Male | Male | Female | Male |
| Age | 50 | 52 | 50 | 43 | 45 | 24 | 51 | 58 |
| IVST (mm) | 11.20 | 10 | 9.1 | 9.9 | 9.9 | 9.9 | 8.00 | 8.30 |
| LVDD (mm) | 48 | 45 | 50 | 75 | 80 | 60 | 41 | 54 |
| LVPW (mm) | 11.20 | 10 | 8.3 | 9.9 | 9.5 | 9.0 | 8.00 | 9.30 |
| EF (%) | 68 | 68 | 69 | 27 | 20 | 69 | 58 | 40 |
| FS (%) | 38 | 38 | 39 | 13 | 10 | 39 | 30 | 20 |
| BNP (0-100 pg/ml) | | | | 902.4 | 540 | | | |
| NT-proBNP (0-300 pg/ml) | 102.2 | Missing | Missing | | | 7194 | 33855 | 1920 |
| NYHA Classification | | | | IV | IV | IV | IV | IV |
| Diagnosis | Normal | Normal | Normal | DCM | DCM | DCM | MS | MS |

IVST: interventricular septum thickness; LVDD: left ventricular diastolic dimension; LVPW: left ventricular posterior wall; EF: ejection fraction;

FS: shortening fraction; BNP: brain natriuretic peptide; NYHA: New York Heart Association; DCM: dilated cardiomyopathy; MS: mitral stenosis.

**Supplementary Table 2. Echocardiographic characterization in a mouse model of HF**

| | LVAWd | LVAWs | LVIDd | LVIDs | LVPWd | LVPWs | EF(%) | FS(%) |
|---|---|---|---|---|---|---|---|---|
| Sham (n=8) | 0.94±0.03 | 1.49±0.04 | 3.22±0.07 | 1.88±0.06 | 0.78±0.03 | 1.24±0.07 | 73.87±1.34 | 41.74±1.21 |
| HF (n=8) | 0.91±0.06 | 1.26±0.06** | 3.52±0.11* | 2.63±0.12*** | 0.80±0.04 | 0.98±0.05* | 51.15±2.48*** | 25.56±1.47*** |

HF: heart failure; LVAWd: diastolic Left ventricle anterior wall; LVAWs: end systolic Left ventricle anterior wall; LVPWd: thickness of diastolic left ventricular posterior wall; LVPWs: thickness of systolic left ventricular posterior wall; LVIDd: left ventricular internal dimension at end-diastole; LVIDs: left ventricular internal dimension at systole; EF: ejection fraction, expressed as percent changes over Sham control; FS: fractional shortening, expressed as percent changes over Sham control; *$P$<0.05 , **$P$<0.01 ,***$P$<0.001 *vs*. Sham control.

**Supplementary Table 3. Echocardiographic characterization in a mouse model of HF with or without Lv-CCRR treatment**

| | LVAWd | LVAWs | LVIDd | LVIDs | LVPWd | LVPWs | EF(%) | FS(%) |
|---|---|---|---|---|---|---|---|---|
| Sham (n=12) | 0.93±0.02 | 1.45±0.04 | 3.28±0.08 | 1.99±0.07 | 0.78±0.04 | 1.22±0.05 | 70.92±1.56 | 39.39±1.30 |
| HF (n=10) | 0.89±0.05 | 1.27±0.05* | 3.46±0.11 | 2.54±0.12*** | 0.80±0.03 | 1.01±0.05** | 53.46±2.50*** | 27.02±1.52*** |
| Lv-CCRR (n=17) | 1.00±0.03 | 1.48±0.05## | 3.52±0.08 | 2.30±0.11 | 0.87±0.02 | 1.24±0.04### | 63.99±2.69## | 34.87±2.20## |
| Lv-NC (n=10) | 1.01±0.06 | 1.38±0.07 | 3.70±0.13** | 2.70±0.11*** | 0.74±0.04* | 1.01±0.03** | 53.24±0.97*** | 27.13±0.56*** |

CCRR: Cardiac Conduction Regulator RNA; HF: heart failure; Lv-CCRR: lentivirus vector carrying the CCRR gene for overexpression. Lv-NC: negative control lentivirus vector; LVAWd: diastolic Left ventricle anterior wall; LVAWs: end systolic Left ventricle anterior wall; LVPWd: thickness of diastolic left ventricular posterior wall; LVPWs: thickness of systolic left ventricular posterior wall; LVIDd: left ventricular internal dimension at end-diastole; LVIDs: left ventricular internal dimension at systole; EF: ejection fraction, expressed as percent changes over HF; FS: fractional shortening, expressed as percent changes over HF. *$P$<0.05 , **$P$<0.01 ,***$P$<0.001 *vs*. Sham control. ##$P$<0.01, ###$P$<0.001 *vs*. HF (heart failure).

**Supplementary Table 4. Echocardiographic characterization in healthy mice with or without Lv-siCCRR treatment**

| | LVAWd | LVAWs | LVIDd | LVIDs | LVPWd | LVPWs | EF(%) | FS(%) |
|---|---|---|---|---|---|---|---|---|
| Sham (n=30) | 0.98±0.02 | 1.56±0.04 | 3.32±0.05 | 1.84±0.05 | 0.79±0.02 | 1.31±0.04 | 76.29±1.23 | 44.38±1.14 |
| Lv-siCCR (n=38) | 0.86±0.02 | 1.26±0.03*** | 3.67±0.05 | 2.62±0.06*** | 0.75±0.03* | 1.06±0.03** | 55.74±1.65*** | 28.83±1.06*** |
| Lv-siNC (n=26) | 0.93±0.03 | 1.45±0.04 | 3.66±0.06 | 2.26±0.08 | 0.81±0.02 | 1.22±0.04 | 68.37±1.75 | 38.09±1.52 |

CCRR: Cardiac Conduction Regulator RNA; Lv-siCCRR: lentivirus vector carrying a CCRR shRNA for silencing endogenous CCRR; ; Lv-siNC: lentivirus vector carrying a scrambled RNA as a negative control for Lv-siCCRR; LVAWd: diastolic Left ventricle anterior wall; LVAWs: end systolic left ventricle anterior wall; LVPWd: thickness of diastolic left ventricular posterior wall; LVPWs: thickness of systolic left ventricular posterior wall; LVIDd: left ventricular internal dimension at end-diastole; LVIDs: left ventricular internal dimension at systole; EF: ejection fraction, expressed as percent changes over Sham; FS: fractional shortening, expressed as percent changes over Sham; **P*<0.05 , ***P*<0.01 ,****P*<0.001 *vs*. Sham control.

**Supplementary Table 5. The Gene-specific primers used for real-time PCR analyses.**

| Gene name | Forward primer (5’→3’) | Reverse primer (5’→3’) |
|---|---|---|
| CCRR (mouse) | CCCTGTTTGAGTTCCTGTCC | CCAAGACCAGAGACCAGAGC |
| GAPDH | AAGAAGGTGGTGAAGCAGGC | TCCACCACCCAGTTGCTGTA |
| CCRR (Human) | GTGCTGCCATCGAAAAATCTG | CTCCCCAGACTCAATGCTTC |
| FD (mouse) | CAGCAGAAGACTGAGCTTTGAA | CTGTCGGGACACTCATCTCA |
| CIP85 (mouse) | GATTCTGTGTACTCCCGCCT | GACCTGTTCATTTAGCCCCA |
